# SGLT2 inhibition potentiates the cardiovascular, renal, and metabolic effects of sGC stimulation in hypertensive rats with prolonged exposure to high-fat diet

**DOI:** 10.1152/ajpheart.00386.2021

**Published:** 2022-02-04

**Authors:** Virginia Reverte, Francisca Rodriguez, Lidia Oltra, Juan M. Moreno, María T. Llinás, Courtney M. Shea, Chad D. Schwartzkopf, Emmanuel S. Buys, Jaime L. Masferrer, F. Javier Salazar

**Affiliations:** ^1^Department of Physiology, School of Medicine, CEIR Mare Nostrum University of Murcia, Murcia, Spain; ^2^Biomedical Research Institute, Murcia, Spain; ^3^Cyclerion Therapeutics, Cambridge, Massachusetts

**Keywords:** high-fat diet, hypertension, insulin resistance, sGC stimulation, SGLT2 inhibition

## Abstract

Prolonged high-fat diet (HFD) accelerates the cardiovascular, renal, and metabolic dysfunction in hypertensive rats with altered renal development (ARDev). Soluble guanylate cyclase (sGC) stimulation or sodium-glucose cotransporter 2 (SGLT2) inhibition may improve cardiovascular, renal, and metabolic function in settings of hypertension and obesity. This study examined whether 6 wk treatment with an SGLT2 inhibitor (empagliflozin, 7 mg/kg/day) enhances the cardiovascular, renal, and metabolic effects of a sGC stimulator (praliciguat, 10 mg/kg/day) in hypertensive rats with ARDev and prolonged exposure to HFD. Arterial pressure (AP), renal vascular resistance (RVR), fat abdominal volume (FAV), insulin resistance, leptin and triglycerides levels, and intrarenal infiltration of inflammatory cells were higher, but cardiac output and creatinine clearance were lower in hypertensive rats (*n* = 15) than in normotensive rats (*n* = 7). Praliciguat administration (*n* = 10) to hypertensive rats reduced (*P* < 0.05) AP, FAV, plasma concentrations of leptin and triglycerides, and increased (*P* < 0.05) cardiac output and creatinine clearance. Empagliflozin administration (*n* = 8) only increased (*P* < 0.05) glucosuria and creatinine clearance and decreased (*P* < 0.05) plasma leptin and triglycerides concentrations in hypertensive rats. Simultaneous administration of praliciguat and empagliflozin (*n* = 10) accelerated the decrease in AP, improved glucose tolerance, reduced (*P* < 0.05) incremental body weight gain, and decreased (*P* < 0.05) insulin resistance index, RVR, and the infiltration of T-CD3 lymphocytes in renal cortex and renal medulla. In summary, the combined administration of praliciguat and empagliflozin leads to a greater improvement of the cardiovascular, renal, and metabolic dysfunction secondary to prolonged exposure to HFD in hypertensive rats with ARDev than the treatment with either praliciguat or empagliflozin alone.

**NEW & NOTEWORTHY** This is the first study, to our knowledge, showing that SGLT2 inhibition potentiates the beneficial cardiovascular, renal, and metabolic effects elicited by sGC stimulation in hypertensive rats with prolonged high-fat diet. The effects of the simultaneous administration of praliciguat and empagliflozin are greater than those elicited by either one alone. The effects of the simultaneous treatment may be related to a greater reduction in the inflammatory status.

## INTRODUCTION

The systemic hypertension and deterioration of renal function secondary to an altered renal development (ARDev) are exacerbated by a prolonged high-fat diet (HFD) (1–3) and are associated with reduced endothelial nitric oxide synthase (eNOS) activity and increased oxidative stress and inflammation (4, 5). It has also been reported that impaired eNOS activity is associated with glucose intolerance and insulin resistance (6) and that a defect in vascular nitric oxide (NO)/cyclic guanosine monophosphate (cGMP) signaling can contribute to the etiology of obesity-related diseases (7). These studies suggest that the cardiovascular, renal, and metabolic dysfunctions elicited by a prolonged HFD in subjects with ARDev may be secondary to changes in the soluble guanylate cyclase (sGC)/NO/cGMP system and, therefore, could be attenuated by administering a sGC stimulator. This hypothesis is supported by studies showing that sGC stimulation has anti-inflammatory (8) and beneficial metabolic effects (9), and that it reduces arterial pressure (AP) and cardiac and renal dysfunction in several models of hypertension (10, 11). However, it is unknown whether prolonged sGC stimulation improves the cardiovascular, renal, and metabolic dysfunctions induced by a prolonged HFD in subjects with ARDev. The first objective of this study was to examine to what extent chronic administration of a sGC stimulator (praliciguat; PRL) reduces AP, renal vasoconstriction, and the renal infiltration of inflammatory cells and also improves glucose homeostasis in hypertensive rats with ARDev and an early and prolonged exposure to HFD.

The inhibition of Na^+^-glucose cotransporter 2 (SGLT2) may induce effects beyond glycemic lowering, including reductions of AP (12), attenuation of inflammatory processes (13), and lower production of reactive oxygen species (14, 15). Considering that the renal effects of SGLT2 inhibitors may be inversely related to the number of intact nephrons (16), the second objective was to evaluate whether SGLT2 inhibition improves the cardiovascular, renal, and metabolic dysfunctions in rats with ARDev. In addition, we also examined the potential additive effects of the simultaneous administration of an SGLT2 inhibitor (empagliflozin; EMPA) and a sGC stimulator on AP, cardiac and renal function, glucose metabolism, fat abdominal volume, and renal infiltration of inflammatory cells in hypertensive rats with ARDev and prolonged exposure to HFD. We hypothesized that the improvement of the cardiovascular, renal, and metabolic dysfunctions elicited by PRL in rats with ARDev and an early and prolonged HFD would be enhanced by the simultaneous EMPA treatment. The results obtained may have clinical implications since overweight and obesity in children and adolescents have risen globally with important public health consequences.

## MATERIAL AND METHODS

All experiments were conducted in male Sprague–Dawley (SD) in accordance with the Directive 2010/63/EU on the protection of animals used for scientific purposes and approved by the University of Murcia Review Committee. All rats had ad libitum access to food and water and were housed in a temperature-controlled room (23°C) with a 12-h:12-h light/dark cycle. Newborn rats were obtained from Murcia University Animal Care Services and treated orally from *postnatal day 1* to *postnatal day 14* with isotonic saline or an AT_1_ receptor antagonist (ARA) (candesartan, 7 mg/kg/day). This ARA treatment induces a 37% reduction in nephron number (17) that is associated with the development of hypertension and a progressive deterioration of renal function (4, 5, 18). It is widely accepted that the administration of a converting enzyme inhibitor or an ARA during the postnatal nephrogenic period significantly reduce nephron endowment (19). After weaning, rats given isotonic saline during the first 2 postnatal wk were fed a normal fat diet (NFD) (Teklad 2014, 13% kcal fat, energy density: 2.9 kcal/g) until the end of the experiment (normotensive group, *n* = 7).

**Figure 1.**
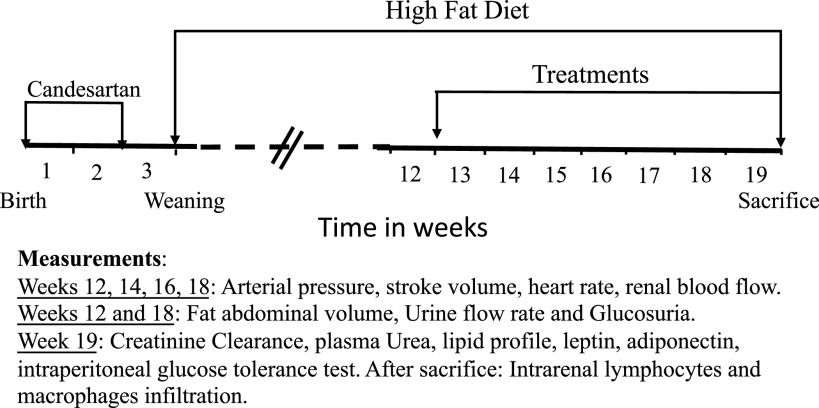
Schematic representation of the experimental design in rats treated with candesartan during the first 2 wk of life. After weaning, rats were exposed to a high-fat diet. Rats were treated with vehicle, empagliflozin, praliciguat, or empagliflozin + praliciguat from 12 to 19 wk of age.

A schematic representation of the experimental design performed in rats treated with the ARA during the first 2 postnatal wk is shown in Fig. 1. They were fed a high-fat diet (HFD; Research Diets, D12492, 60% kcal fat, energy density: 5.21 kcal/g) until the end of the experiment. At *week 13*, hypertensive rats with HFD were randomly assigned to four different groups: without any treatment (vehicle group; *n* = 15); treated with a sodium-glucose cotransporter 2 inhibitor (EMPA, 7 mg/kg/day) in drinking water (EMPA group; *n* = 8); treated with an sGC stimulator (PRL, 10 mg/kg/day) formulated in the HFD (Research Diets, D18071902) (PRL group; *n* = 10); or treated with EMPA and PRL as aforementioned (EMPA + PRL group, *n* = 10). PRL is a highly selective sGC stimulator with rapid absorption, high bioavailability, and nonrenal clearance (20, 21) that improves cardiorenal damage by reducing AP and several markers of inflammation and fibrosis (22). The sGC stimulator, PRL binds to and stimulates heme-containing NO-responsive sGC. Therefore, PRL acts with NO and can maintain or amplify endogenous NO signaling.

Body weight and food intake were recorded weekly from 12 wk to the end of the study. Arterial pressure, stroke volume (SV), heart rate (HR), and renal blood flow (RBF) were measured at *weeks 12*, *14*, *16*, and *18* of age in isoflurane anesthetized rats (23). Cardiac output (CO) values were calculated from SV and HR. Total peripheral resistance (TPR) was calculated from mean AP (MAP) and CO values. Renal vascular resistance (RVR) was calculated from MAP and RBF values. Fat abdominal volume (FAV), urine flow rate (UFR), and glucosuria were measured at 12 and 18 wk of age. Urine samples were collected the 2nd day that rats were housed individually during 2 days in metabolic cages with the rat chow (NFD, HFD, or HFD+PRL) and drinking water (tap or with EMPA). Creatinine clearance and plasma urea and triglycerides (TG) concentrations were measured at 19 wk of age. An intraperitoneal glucose tolerance test was performed at *week 19*, as previously described (24, 25). Rats were anesthetized (Pentothal, Abbott) and blood withdrawn by cardiac puncture after these measurements were completed. Plasma samples were stored at −80°C. After that, rats were perfused with saline and formalin (30% PBS) to examine the T-CD3 lymphocytes and CD68 macrophages infiltration in the renal cortex and renal medulla (2).

### Arterial Pressure Measurement

AP was measured by plethysmography (CODA, Kent Scientific Corporation, Connecticut) under superficial anesthesia with isoflurane to avoid the stress during the inflation-deflation cycles in the rat tail. The systolic and mean AP values obtained by plethysmography are highly correlated with those obtained in conscious freely moving rats with intra-arterial catheters (18).

### Ultrasound Measurements

Ultrasound measurements were performed, as previously described (23), in isoflurane anesthetized rats on a heated platform to maintain rectal temperature at 37°C. SV, HR, and RBF were evaluated using a high-resolution micro-ultrasound system (Vevo 3100, VisualSonics, Toronto, Canada) and two transducers: MX250 (axial resolution: 50 µm; frequency: 25 MHz) and MX400 (axial resolution: 75 µm; frequency: 40 MHz). B-mode and M-mode echocardiographic evaluations were performed using the 25 MHz transducer. B-mode was activated to visualize the heart, then M-mode was activated to obtain SV, HR, and CO values from at least three consecutive cardiac cycles. Blood flow was measured in the left kidney using 40-MHz probe. B-mode was activated to visualize the renal artery and its diameter measured tracing a line between the internal opposite sides of the arterial wall in frozen images (23). Five measurements were obtained in each image to get the arterial diameter. Velocity time integral (VTI) was obtained by outlining five consecutive heartbeat cycles and the integral under the curve calculated. The RBF was calculated from RBF = HR × VTI × π*r*^2^, where *r* is the vessel radius.

### Creatinine Clearance

Glomerular filtration rate (GFR) was determined by the endogenous creatinine clearance at the end of the experiment. This method has been used previously by our group in conscious animals (18), and the GFR values found were similar to those obtained in anesthetized rats using the [^3^H] inulin clearance (4).

### Intraperitoneal Glucose Tolerance Test

Glucose concentrations (mg/dL) were measured by glucometer in a drop of blood (∼35 µL) from the tail vein before (0 min) and after (30, 60, 90, and 120 min) an intraperitoneal glucose load (2 g/kg body wt) (24). Blood samples (∼150 µL) were collected from the tail vein before (*t*0) and 30 min after (*t*30) the glucose challenge to measure insulin concentrations (ng/mL).

### Biochemical Assays

Creatinine, urea, TG, HDL and LDL cholesterol, and glucose concentrations were measured by a BioSystems (A25) analyzer. Plasma leptin and adiponectin levels were quantified by ELISA kits (R&D System, Minneapolis, MN). Plasma insulin concentrations were quantified by ELISA kit (EMD Millipore, Billerica, MA). Insulin resistance (IR) was calculated by the homeostatic model assessment index as homeostatic model assessment for insulin resistance (HOMA-IR) [insulin (mUI/mL) × glucose (mmol/L)/22.5] as in previous studies (25).

### Fat Abdominal Volume

Fat abdominal volume (FAV) was examined by computerized tomography (CT) as an index of abdominal obesity. Images were obtained in anesthetized rats (isoflurane) using the Albira CT system (Bruker Molecular Imaging, Woodbridge, CT) (26) and examined using the PMOD (PMOD Technologies, Zurich, Switzerland) software. Images were segmented according to tissue density, first for total abdominal volume and then for fat volume (26). FAV calculations may include visceral and subcutaneous fat because CT does not differentiate between visceral and subcutaneous fat. However, since the contour of the abdominal zone to be examined was manually drawn inside the abdominal wall, it is considered that the image obtained with the CT mainly shows the visceral fat in the abdominal area. Computerized tomography was used in our study because the objective was to examine the evolution from 12 to 18 mo of age.

### Immunohistopathology

Immunohistopathologic analyses of T-CD3 lymphocytes and CD68 macrophages infiltrates in the renal cortex and renal medulla were carried out as described (2, 26), on 3-µm-thick sections from formalin-fixed and paraffin-embedded samples obtained from each group of rats. Polyclonal rabbit anti-T CD3 (dilution 1:500, Dako, A0452, Barcelona, Spain) or monoclonal rat anti-CD68 (dilution 1:100, Merck, MAB1435, Madrid, Spain) were first used. Then, sections were incubated with a secondary anti-rabbit (Dako) or anti-rat (Vector, Madrid, Spain). Positive reaction was identified as described (2, 26). Negative controls, obtained by incubating the samples only with dilution agent instead with the primary antibodies were also examined. For T-CD3 and CD68 quantification, the average number of positive cells in 10 high-power fields (×400) were calculated in kidney samples of each rat. For immunophenotypic determinations, a standard direct-light microscope (Zeiss Axio Scope A10, Carl Zeiss, Jenna, Germany) with a digital camera (AxioCam 506 Color, Carl Zeiss) and a specialized digital analysis software (Zeiss Zen Ver. 3.0, Carl Zeiss, and ImageJ Ver. 1.8.0_172, NHI) were employed.

### Statistical Analysis

Results are expressed as means ± SE. All data were analyzed using GraphPad Prism software, and statistical significance was defined as *P* < 0.05. Time course of each treatment was evaluated using repeated measures one-way ANOVA with Holm–Sidak’s multiple comparisons test. Two-way ANOVA were used to compare time course data among the different experimental groups, with Tukey’s multiple comparisons test to evaluate the effect of different treatments at each time point. Comparisons between data obtained in normotensive and hypertensive rats at 12 wk of age, and at the end of the study (*week 19*) between each experimental group were analyzed by unpaired *t* test.

## RESULTS

### Body Weight, Food Intake, Urine Volume, Glucosuria, and Sodium Excretion

**Figure 2.**
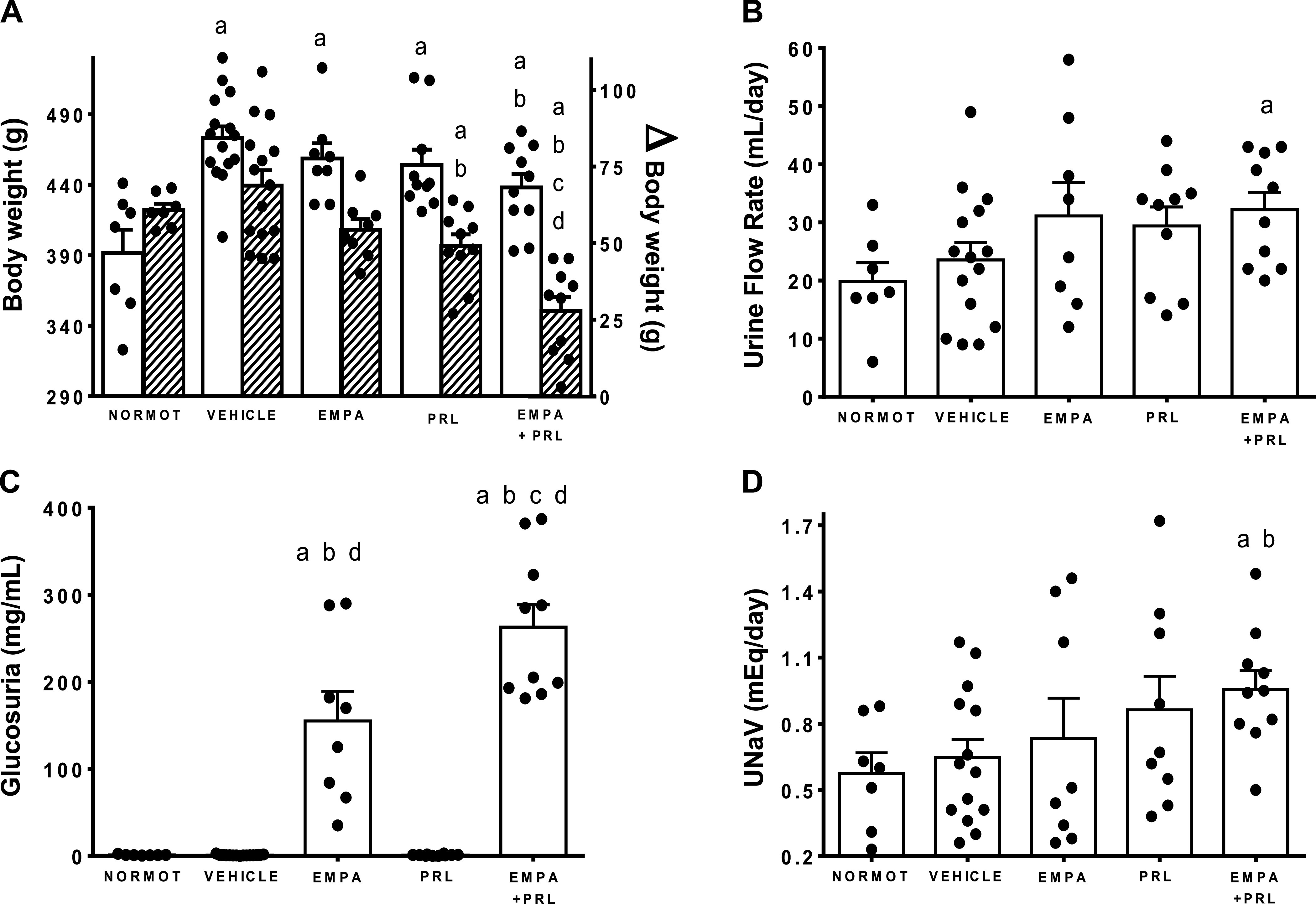
Body weight (*A*, white bars), urine flow rate (*B*), glucose excretion (*C*), and urinary sodium excretion (UNaV; *D*) in normotensive rats (NORMOT) (*n* = 7) and in hypertensive rats treated with vehicle (*n* = 15), empagliflozin (EMPA) (*n* = 8), praliciguat (PRL) (*n* = 10), or empagliflozin + praliciguat (EMPA+PRL) at 18 wk of age. The incremental body weight from 12 to 18 wk of age is shown in *A* (shaded bars). Bars show means ± SE. Differences between groups were assessed with the use of unpaired *t* test. ^a^*P* < 0.05 vs. normotensive; ^b^*P* < 0.05 vs. vehicle; ^c^*P* < 0.05 vs. empagliflozin; and ^d^*P* < 0.05 vs. praliciguat.

Body weight was greater (*P* < 0.0001) in hypertensive rats on HFD (406 ± 4 g) than in normotensive rats (331 ± 5 g) at 12 wk of age and remained elevated at 18 wk of age in each group of rats with HFD after administering EMPA and/or PRL (Fig. 2). However, the incremental change in body weight from 12 to 18 wk of age was greater in hypertensive rats treated with vehicle (69 ± 5 g) than in those treated PRL (49 ± 4 g, *P* = 0.009) or EMPA+PRL (28 ± 5 g, *P* < 0.0001). The incremental change in body weight was lower in rats treated with EMPA+PRL than in those treated with either EMPA (*P* = 0.0005) or PRL (*P* = 0.00023) alone (Fig. 2). Food intake was greater (*P* < 0.0001) in normotensive (20.3 ± 0.7 g/day) than in hypertensive (14.6 ± 0.4 g/day) rats at 12 wk of age and did not change in any group throughout the experimental period. UFR did not change from 12 to 18 wk of age in normotensive rats or in hypertensive rats treated with vehicle or PRL but increased (*P* < 0.0001) in those treated with EMPA (Δ = 12.5 ± 2.2 mL/day) or EMPA+PRL (Δ = 14.5 ± 1.1 mL/day). UFR was variable but higher (*P* = 0.0079) in EMPA+PRL-treated rats than in normotensive rats at 18 wk of age (Fig. 2). Glucosuria was similar in normotensive rats (1.03 ± 0.22 mg/day), in hypertensive rats treated with vehicle (0.87 ± 0.16 mg/day) and in rats treated with PRL (0.89 ± 0.27 mg/day). Glucosuria was higher (*P* < 0.0001) in rats treated with EMPA (155 ± 34 mg/day) and in those treated with EMPA+PRL (263 ± 26 mg/day) than in normotensive rats, being higher (*P* = 0.020) in rats with the combined treatment than in rats only treated with EMPA (Fig. 2). Urinary sodium excretion (UNaV) was similar at 18 wk of age in normotensive rats (0.57 ± 0.09 mEq/day) and hypertensive rats treated with vehicle (0.65 ± 0.08 mEq/day), EMPA (0.73 ± 0.18 mEq/day) or PRL (0.86 ± 0.15 mEq/day). However, UNaV was greater (*P* = 0.0178) in rats treated with EMPA+PRL (0.96 ± 0.08 mEq/day) than in vehicle treated rats (Fig. 2).

### Arterial Pressure, Cardiac and Total Peripheral Resistance Changes

**Figure 3.**
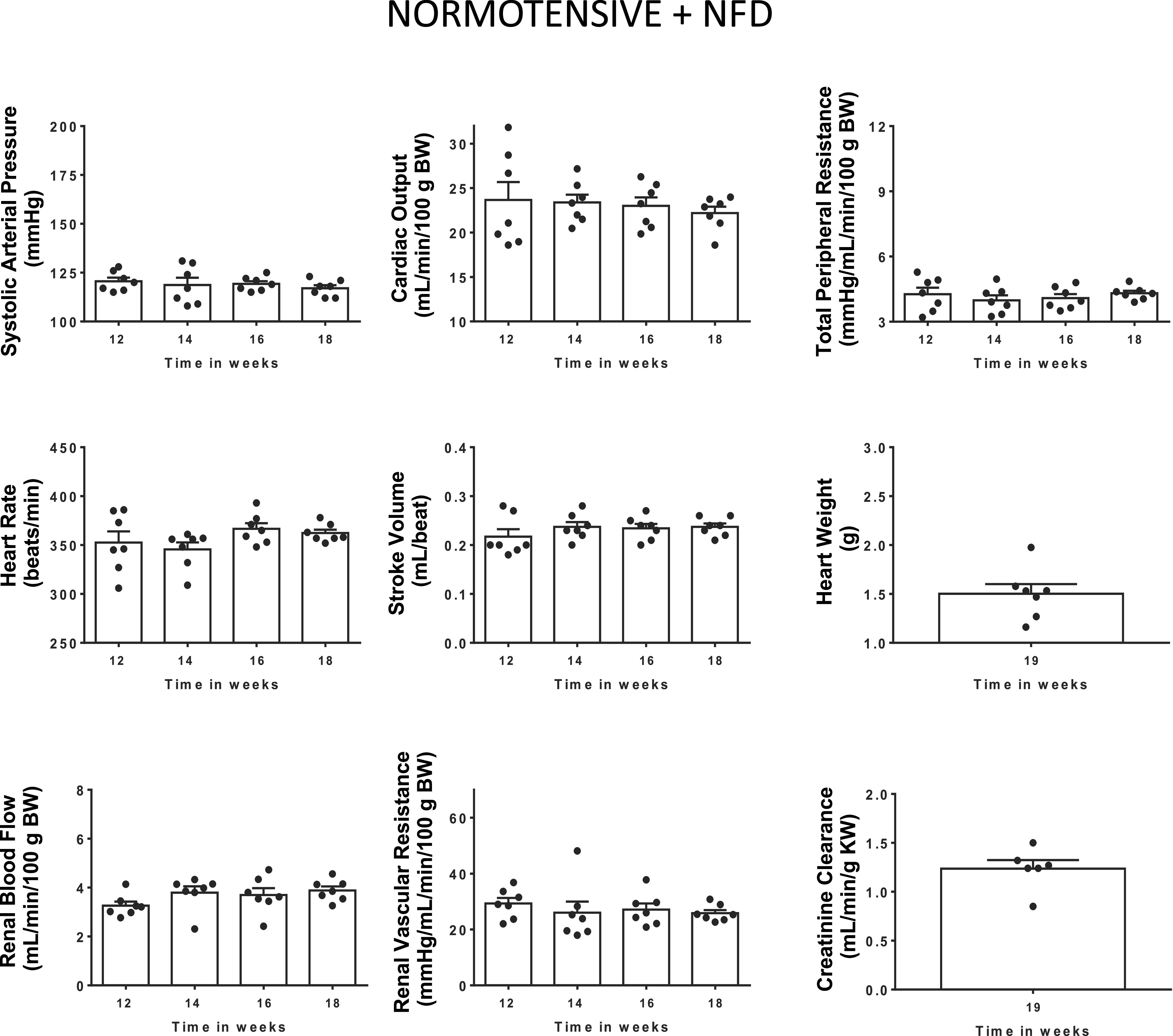
Systolic arterial pressure, cardiac output, total peripheral resistance, heart rate, stroke volume, renal blood flow, and renal vascular resistance from 12 to 18 wk of age, and heart weight, and endogenous creatinine clearance at 18 wk of age in normotensive rats with normal fat diet (NFD) from weaning (*n* = 7). Bars show means ± SE. Differences vs. basal period (*week 12*) were assessed with the use of one-way ANOVA for repeated measures with Holm–Sidak’s multiple comparisons test.

**Figure 4.**
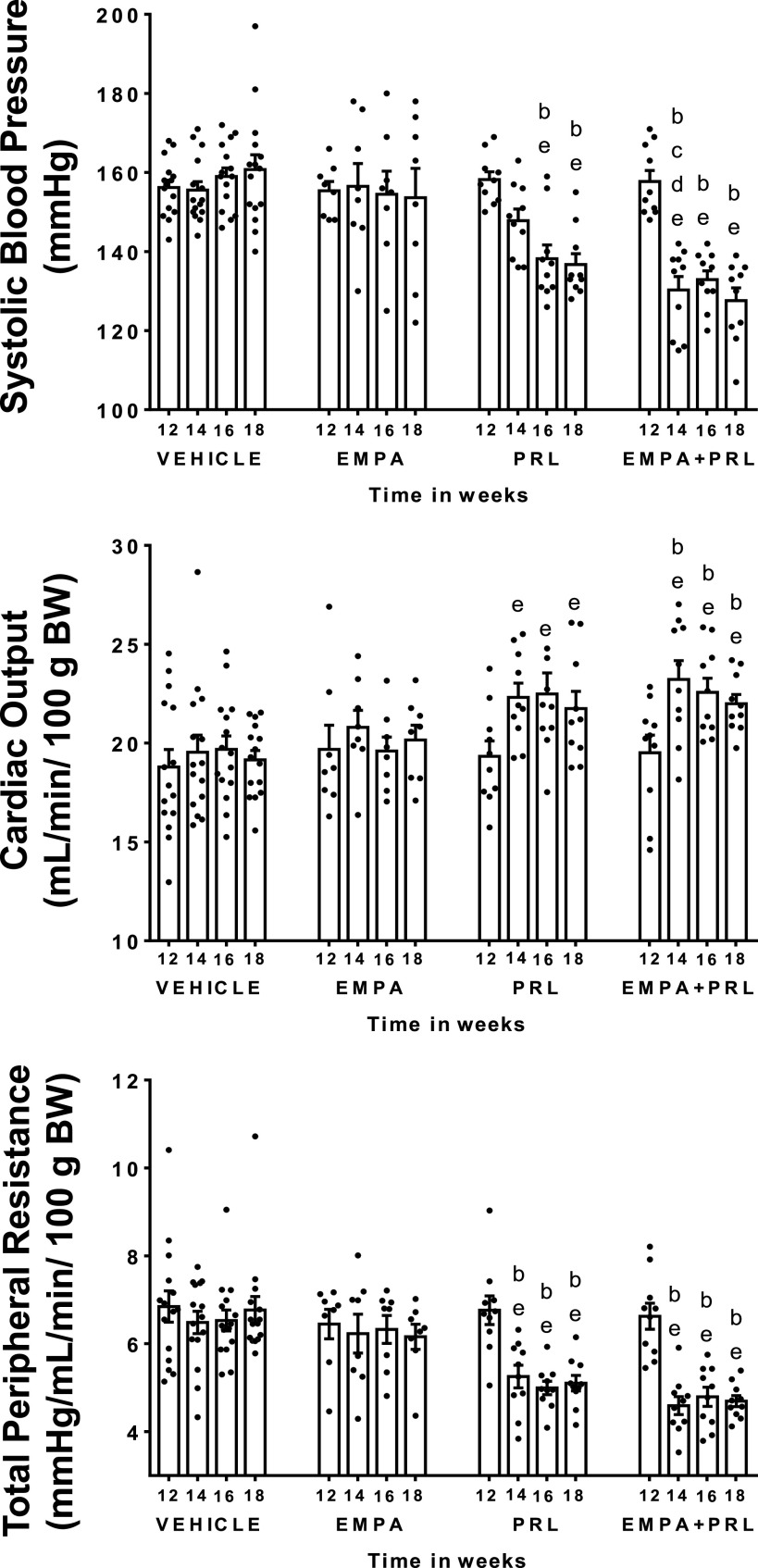
Systolic arterial pressure, cardiac output, and total peripheral resistance in high-fat diet (HFD)-fed hypertensive rats treated from 12 to 18 wk of age with vehicle (*n* = 15), empagliflozin (EMPA) (*n* = 8), praliciguat (PRL) (*n* = 10), or empagliflozin + praliciguat (EMPA+PRL) (*n* = 10). Bars show means ± SE. Differences vs. basal period (*week 12*) were assessed with the use of one-way ANOVA for repeated measures with Holm–Sidak’s multiple comparisons test. Differences between groups were assessed with the use of two-way ANOVA with Tukey’s multiple comparisons analysis. ^b^*P* < 0.05 vs. vehicle; ^c^*P* < 0.05 vs. empagliflozin; ^d^*P* < 0.05 vs. praliciguat; and ^e^*P* < 0.05 vs. *week 12*.

**Figure 5.**
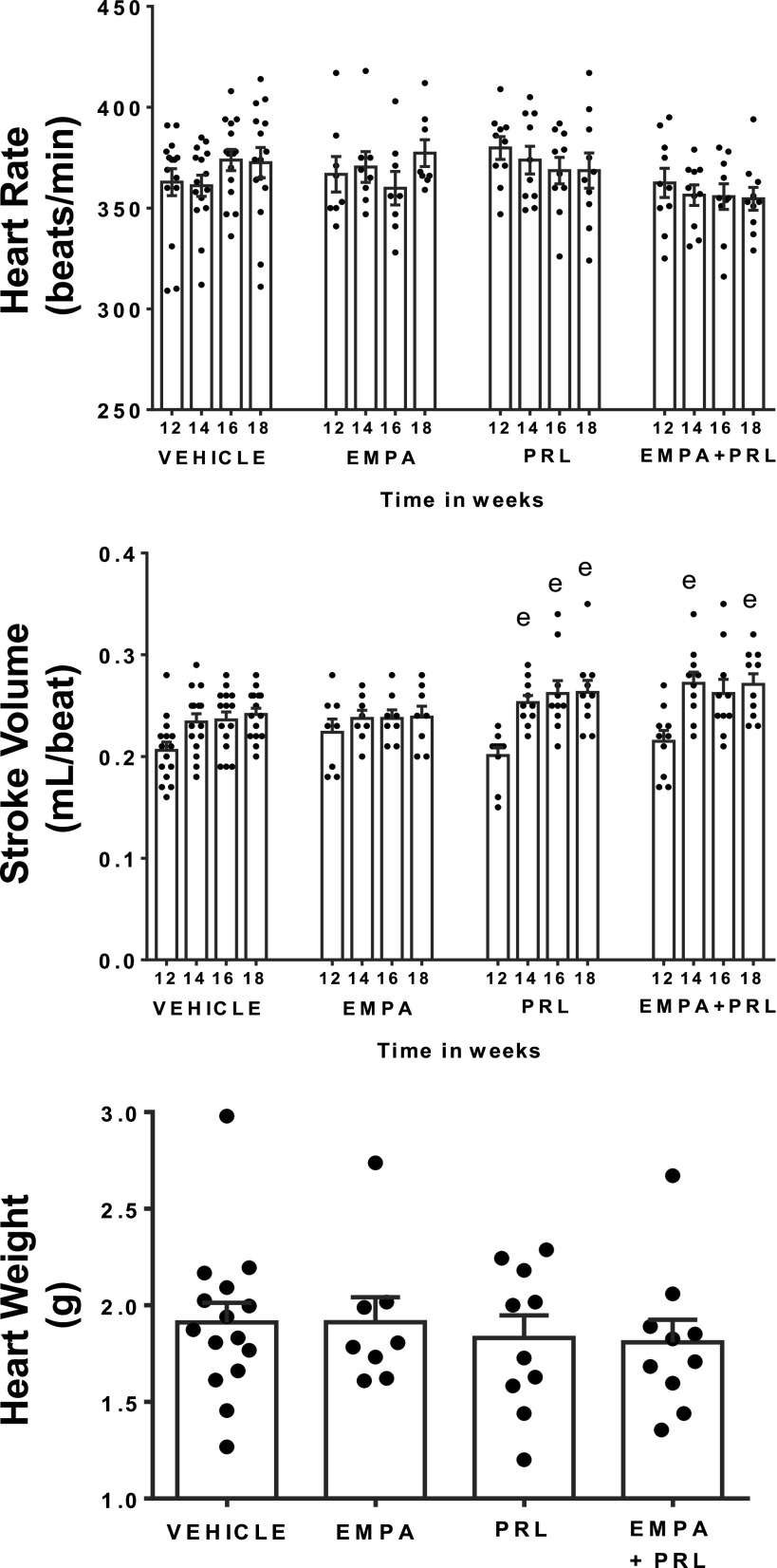
Heart rate and stroke volume from 12 to 18 wk of age and heart weight at 19 wk of age in high-fat diet (HFD)-fed hypertensive rats treated with vehicle (*n* = 14 or 15), empagliflozin (EMPA) (*n* = 8), praliciguat (PRL) (*n* = 10) or EMPA + PRL (*n* = 10). Bars show means ± SE. Differences vs. basal period (*week 12*) were assessed with the use of one-way ANOVA for repeated measures with Holm–Sidak’s multiple comparison test. Differences between groups were assessed with the use of two-way ANOVA with Tukey’s multiple comparisons analysis. ^e^*P* < 0.05 vs. *week 12*.

Systolic arterial pressure (SAP) was higher (*P* < 0.0001) in hypertensive (157 ± 1 mmHg) than in normotensive (121 ± 2 mmHg) rats at 12 wk of age. SAP, CO, TPR, HR, and SV did not change from 12 to 18 wk of age in normotensive rats with NFD (Fig. 3). Similarly, SAP did not change during this experimental period in hypertensive rats treated with vehicle or EMPA (Fig. 4). However, SAP decreased (*P* = 0.0002) after PRL (Δ = 22 ± 3 mmHg) or EMPA+PRL (Δ = 29 ± 4 mmHg) treatment (Fig. 4). The decrease in SAP was significant (*P* = 0.001) after the 2nd week of simultaneous EMPA+PRL treatment but not of PRL treatment alone. No significant changes in HR were observed from 12 to 18 mo of age in any group (Fig. 5). Stroke volume did not change in hypertensive rats treated with vehicle or EMPA but increased when rats were treated with PRL (*P* = 0.032) or EMPA+PRL (*P* = 0.010) (Fig. 5). CO was lower (*P* < 0.0001) in hypertensive (19 ± 1 mL/min/100 g body wt) than in normotensive (24 ± 2 mL/min/100 g body wt) rats at 12 wk of age and did not change significantly throughout the study in hypertensive rats treated with vehicle or EMPA (Fig. 4). However, CO increased after six weeks of PRL (*P* = 0.0263) or EMPA+PRL (*P* = 0.0239) treatment to 22 ± 1 mL/min/100 g body wt (Fig. 4). TPR was greater (*P* = 0.001) in hypertensive (6.7 ± 0.2 mmHg/mL/min/100g body wt) than in normotensive (4.3 ± 0.3 mmHg/mL/min/100g body wt) rats at 12 wk of age and did not change significantly from 12 to 18 wk of age in hypertensive rats treated with vehicle or EMPA (Fig. 4). Six weeks of treatments with PRL alone or EMPA+PRL led to a significant decrease (*P* < 0.05) in TPR (5.1 ± 0.2 and 4.7 ± 0.1 mmHg/mL/min/100 g body wt, respectively). Heart weight was greater (*P* = 0.0083) in vehicle-treated hypertensive rats (1.91 ± 0.10 g) than in normotensive rats (1.50 ± 0.10 g) and was not significantly altered by EMPA, PRL or EMPA+PRL treatment (Figs. 3 and 5).

### Renal Changes

**Figure 6.**
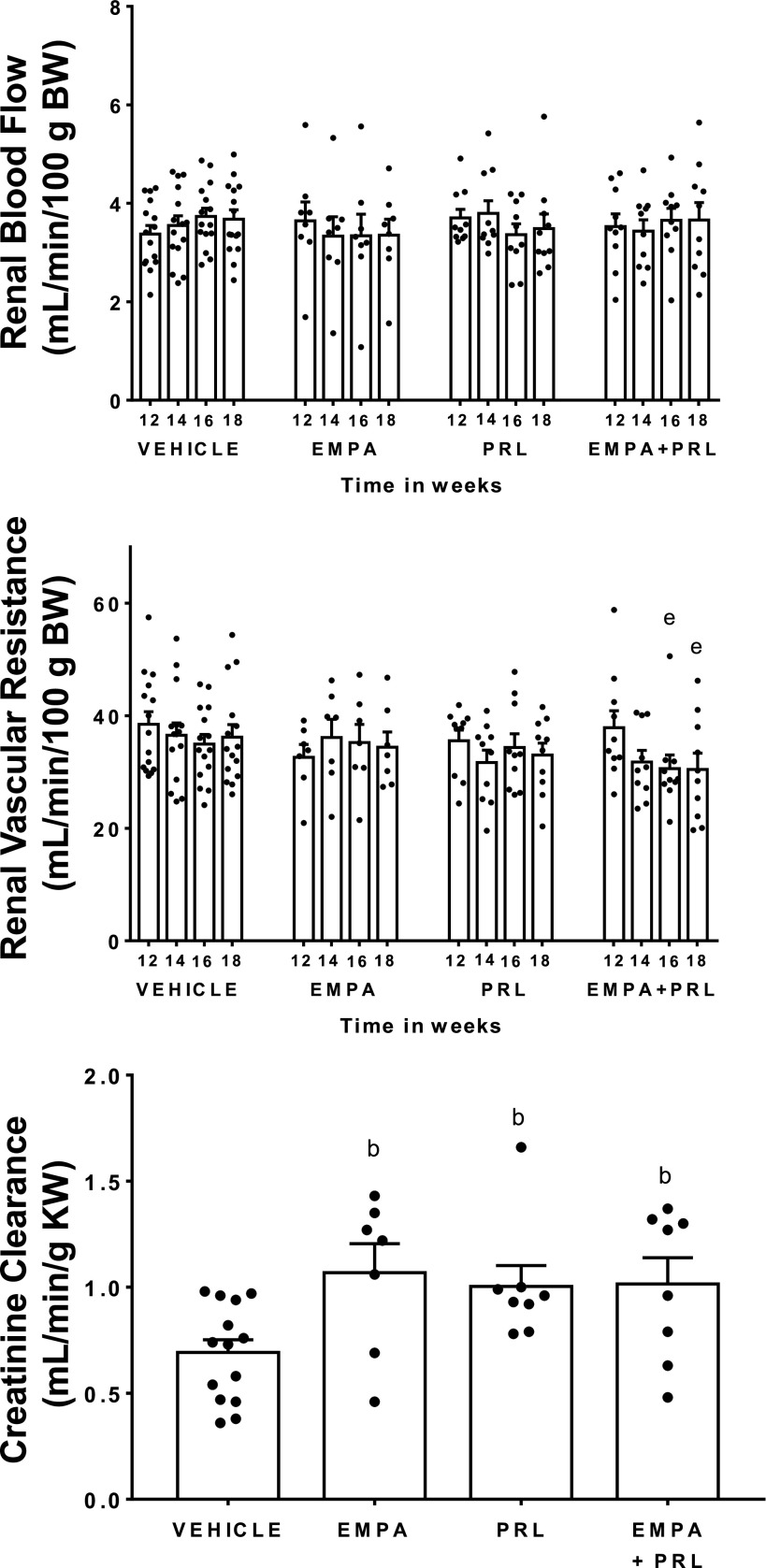
Renal blood flow and renal vascular resistance from *weeks 12* to *18* of age and creatinine clearance at *week 19* of age in high-fat diet (HFD)-fed hypertensive rats treated with vehicle (*n* = 14 or 15), empagliflozin (EMPA) (*n* = 7 or 8), praliciguat (PRL) (*n* = 8–10), or empagliflozin + praliciguat (EMPA+PRL) (*n* = 8–10). Bars show means ± SE. Differences vs. basal period (*week 12*) were assessed with the use of one-way ANOVA for repeated measures with Holm–Sidak’s multiple comparisons test. Differences between groups were assessed with the use of two-way ANOVA with Tukey’s multiple comparisons analysis. ^b^*P* < 0.05 vs. vehicle; and ^e^*P* < 0.05 vs. *week 12*.

**Figure 7.**
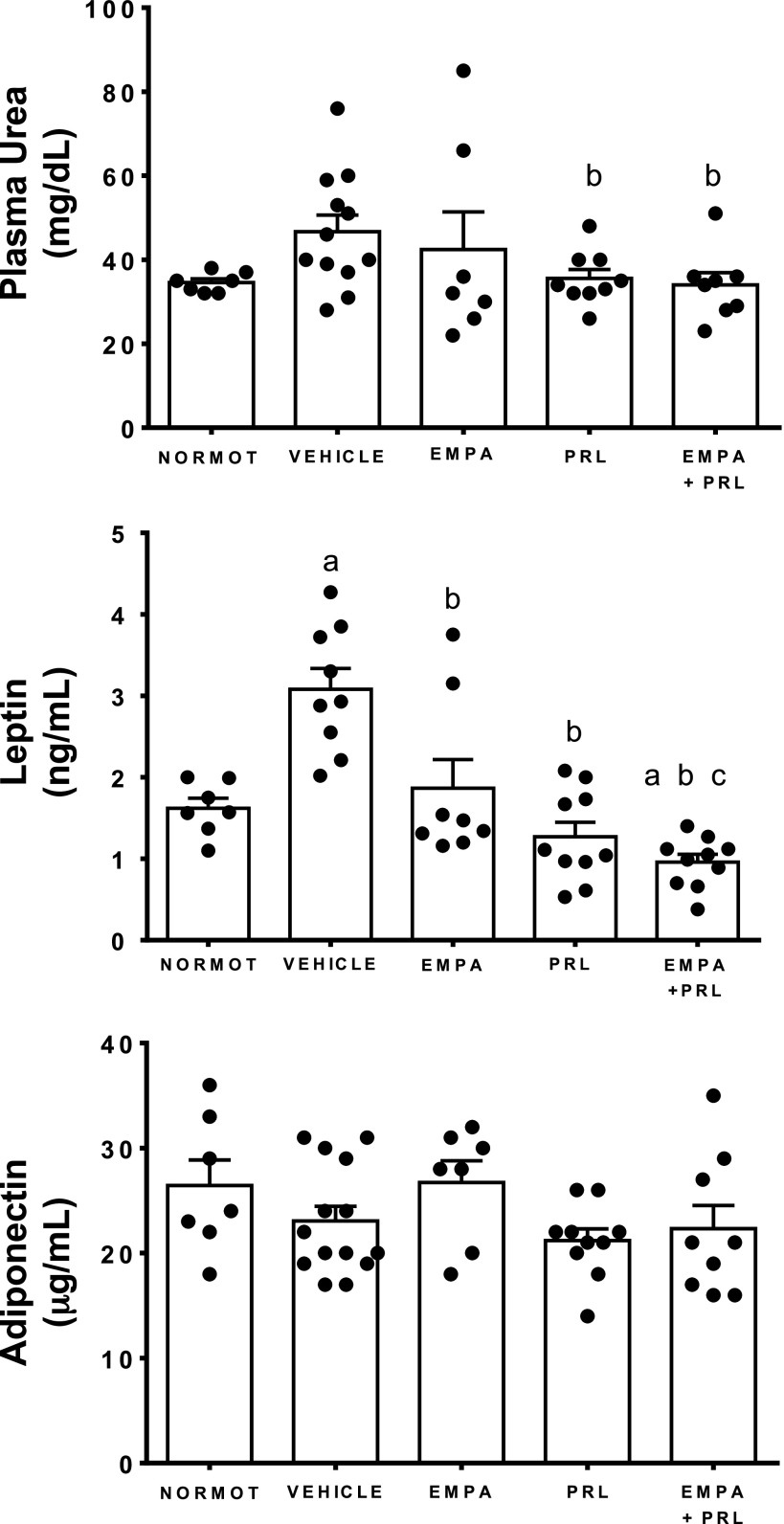
Plasma urea, leptin, and adiponectin concentrations in normotensive rats (NORMOT) (*n* = 7) and in hypertensive rats treated with vehicle (*n* = 14), empagliflozin (EMPA) (*n* = 7), praliciguat (PRL) (*n* = 9 or 10), or empagliflozin + praliciguat (EMPA+PRL) (*n* = 8 or 9) at 19 wk of age. Bars show means ± SE. Differences between groups were assessed with the use of unpaired *t* test. ^a^*P* < 0.05 vs. normotensive; ^b^*P* < 0.05 vs. vehicle; and ^c^*P* < 0.05 vs. empagliflozin.

Renal blood flow was similar in normotensive and in hypertensive rats at 12 wk of age and did not change throughout the study in any experimental group (Figs. 3 and 6). However, RVR was greater (*P* = 0.048) in hypertensive (38 ± 2 mmHg/mL/min/100g body wt) than in normotensive (31 ± 2 mmHg/mL/min/100g body wt) rats at that age. Six weeks of treatments with vehicle, EMPA, or PRL alone did not modify RVR but treatment with EMPA+PRL led to an 18 ± 5% decrease (*P* = 0.030) in RVR, that was significant after 4 wk of their simultaneous administration (Fig. 6). At the end of the experiment, creatinine clearance was lower (*P* = 0.0006) in hypertensive rats treated with vehicle (0.69 ± 0.06 mL/min/g kw) than in normotensive rats (1.24 ± 0.09 mL/min/g kw) (Figs. 3 and 6). Treatment of hypertensive rats with EMPA, PRL, or EMPA+PRL induced an increase (*P* < 0.05) in creatinine clearance (1.07 ± 0.15, 1.00 ± 0.11, and 1.01 ± 0.12 mL/min/g kw, respectively) (Fig. 6). Plasma urea levels were greater (*P* = 0.036) in hypertensive rats treated with vehicle (47 ± 4 mg/mL) than in normotensive rats (35 ± 1 mg/mL). Plasma urea levels were not modified by EMPA treatment (42 ± 9 mg/mL) but decreased after PRL (36 ± 2 mg/mL, *P* = 0.037) or EMPA+PRL treatments (34 ± 3 mg/mL, *P* = 0.032) (Fig. 7).

### Changes in Plasma Levels of Leptin and Adiponectin, Fat Abdominal Volume, and Lipid Profile

**Figure 8.**
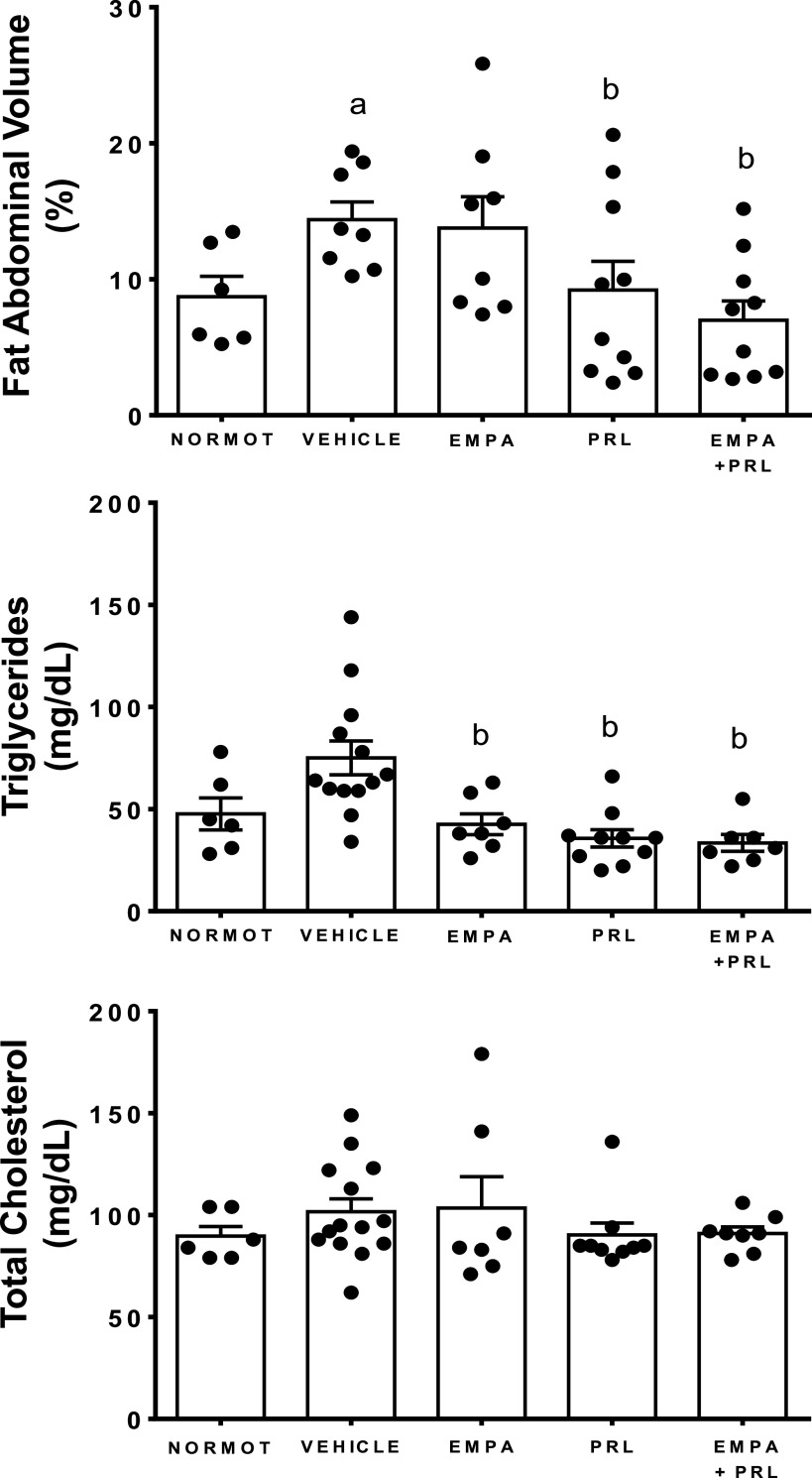
Fat abdominal volume and plasma triglycerides and cholesterol concentrations in normotensive rats (NORMOT) (*n* = 7) and in hypertensive rats treated with vehicle (*n* = 9–13), empagliflozin (EMPA) (*n* = 7 or 8), praliciguat (PRL) (*n* = 10) or empagliflozin + praliciguat (EMPA+PRL) (*n* = 8–10), at 19 wk of age. Bars show means ± SE. Differences between groups were assessed with the use of unpaired *t* test. ^a^*P* < 0.05 vs. normotensive; and ^b^*P* < 0.05 vs. vehicle.

Plasma leptin levels were greater in hypertensive rats with HFD treated with vehicle (3.1 ± 0.3 ng/mL) than in normotensive rats (1.6 ± 0.1 ng/mL, *P* = 0.0003) and in hypertensive rats treated with EMPA (1.9 ± 0.4 ng/mL, *P* = 0.0206), PRL (1.3 ± 0.2 ng/mL, *P* < 0.0001), and EMPA+PRL (1.0 ± 0.1 ng/mL, *P* < 0.0001). Leptin levels were lower (*P* = 0.015) in rats treated with EMPA+PRL than in EMPA-treated rats (Fig. 7). Plasma adiponectin concentration was similar in normotensive rats (26 ± 3 ng/mL) and hypertensive rats treated with vehicle (23 ± 2 µg/mL), EMPA (27 ± 2 µg/mL), PRL (21 ± 1 ng/mL), or EMPA+PRL (22 ± 2 µg/mL) (Fig. 7). FAV was greater (*P* < 0.0001) in hypertensive (14.1 ± 0.6%) than in normotensive (6.6 ± 1.1%) rats at 12 wk of age. Treatment with vehicle (14.4 ± 1.3%) or EMPA (13.8 ± 2.3%) did not induce significant changes in FAV at the end of experimental period. However, FAV was lower in rats treated with PRL (9.2 ± 2.1%, *P* = 0.0335) or EMPA+PRL (6.9 ± 1.4%, *P* = 0.0017) than in vehicle-treated rats (Fig. 8). Plasma TG concentration was greater in hypertensive rats treated with vehicle (75 ± 8 mg/dL) than in normotensive rats (48 ± 8 mg/dL, *P* = 0.0284) and hypertensive rats treated with EMPA (43 ± 5 mg/dL, *P* = 0.0145), PRL (36 ± 4 mg/dL, *P* = 0.0009), or EMPA+PRL (33 ± 4 mg/dL, *P* = 0.0024) (Fig. 8). No significant differences in total cholesterol (Fig. 8) and HDL or LDL cholesterol were found between groups.

### Changes in Glucose Tolerance and Insulin Resistance

**Figure 9.**
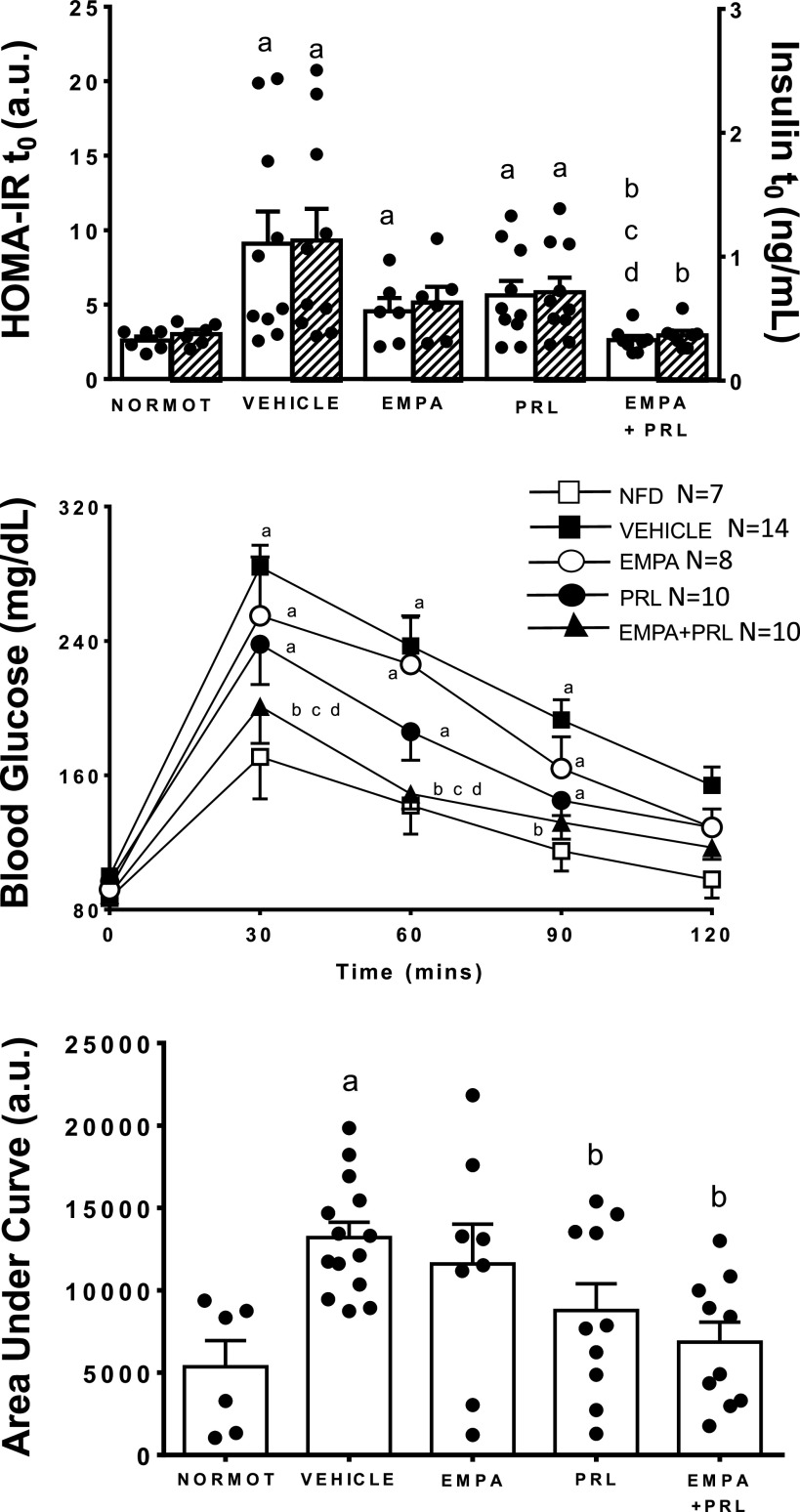
Homeostatic model assessment for insulin resistance (HOMA-IR) (white bars) and plasma insulin levels before a glucose challenge (*t*_0_) (shaded bars), and blood glucose time course and area under the curve after a glucose overdose, in normotensive rats (NORMOT) (*n* = 7) and in hypertensive rats treated with vehicle (*n* = 11–14), empagliflozin (EMPA) (*n* = 7 or 8), praliciguat (PRL) (*n* = 8–10) or empagliflozin + praliciguat (EMPA+PRL) (*n* = 8–10). Bars show means ± SE. Differences between groups were assessed with the use of two-way ANOVA with Tukey’s multiple comparisons analysis. ^a^*P* < 0.05 vs. normotensive; ^b^*P* < 0.05 vs. vehicle; ^c^*P* < 0.05 vs. empagliflozin; and ^d^*P* < 0.05 vs. praliciguat.

Fasting glucose and insulin levels were greater (*P* = 0.0001) in hypertensive rats treated with vehicle (100 ± 2 mg/mL and 1.13 ± 0.26 ng/mL, respectively) than in normotensive rats (87 ± 2 mg/mL and 0.40 ± 0.04 ng/mL, respectively) (Fig. 9). Fasting glucose levels decreased (*P* < 0.05) after EMPA (92 ± 2 mg/mL, *P* = 0.003) or EMPA+PRL (89 ± 2 mg/mL, *P* = 0.0002) treatments but not after PRL treatment alone (97 ± 2 mg/mL). Fasting plasma insulin levels did not change significantly in hypertensive rats treated with EMPA (0.63 ± 0.11 ng/mL) or PRL (0.71 ± 0.12 ng/mL) alone but decreased (*P* = 0.0022) after the simultaneous EMPA and PRL treatment (0.37 ± 0.03 ng/mL). HOMA-IR, calculated with these plasma glucose and insulin levels, was greater in hypertensive rats treated with vehicle (9.1 ± 2.2) than in normotensive rats (2.8 ± 0.3) (Fig. 9). Treatment with EMPA+PRL (2.6 ± 0.3), but not with EMPA (4.6 ± 0.9) or PRL (5.6 ± 1.0) alone, reduced HOMA-IR to the levels detected in normotensive rats. Figure 9 also shows the changes in glucose levels and the area under curve (AUC) of these changes during 120 min after glucose administration in each experimental group. Glucose concentration increased more (*P* < 0.05) after 30 and 120 min of glucose challenge in hypertensive rats treated with vehicle (284 ± 13 and 154 ± 11 mg/dL, respectively) than in normotensive rats (171 ± 25 and 98 ± 11 mg/dL, respectively) (Fig. 9). The AUC was also greater (*P* = 0.0003) in hypertensive rats treated with vehicle (13,200 ± 928) than in normotensive rats (5,310 ± 1,611). The AUC decreased after PRL administration (8,769 ± 1,821, *P* = 0.0193) and after the simultaneous administration of EMPA and PRL (6,846 ± 1,220, *P* = 0.0004) (Fig. 9).

### Lymphocytes and Macrophages Infiltration in Renal Tissue

**Figure 10.**
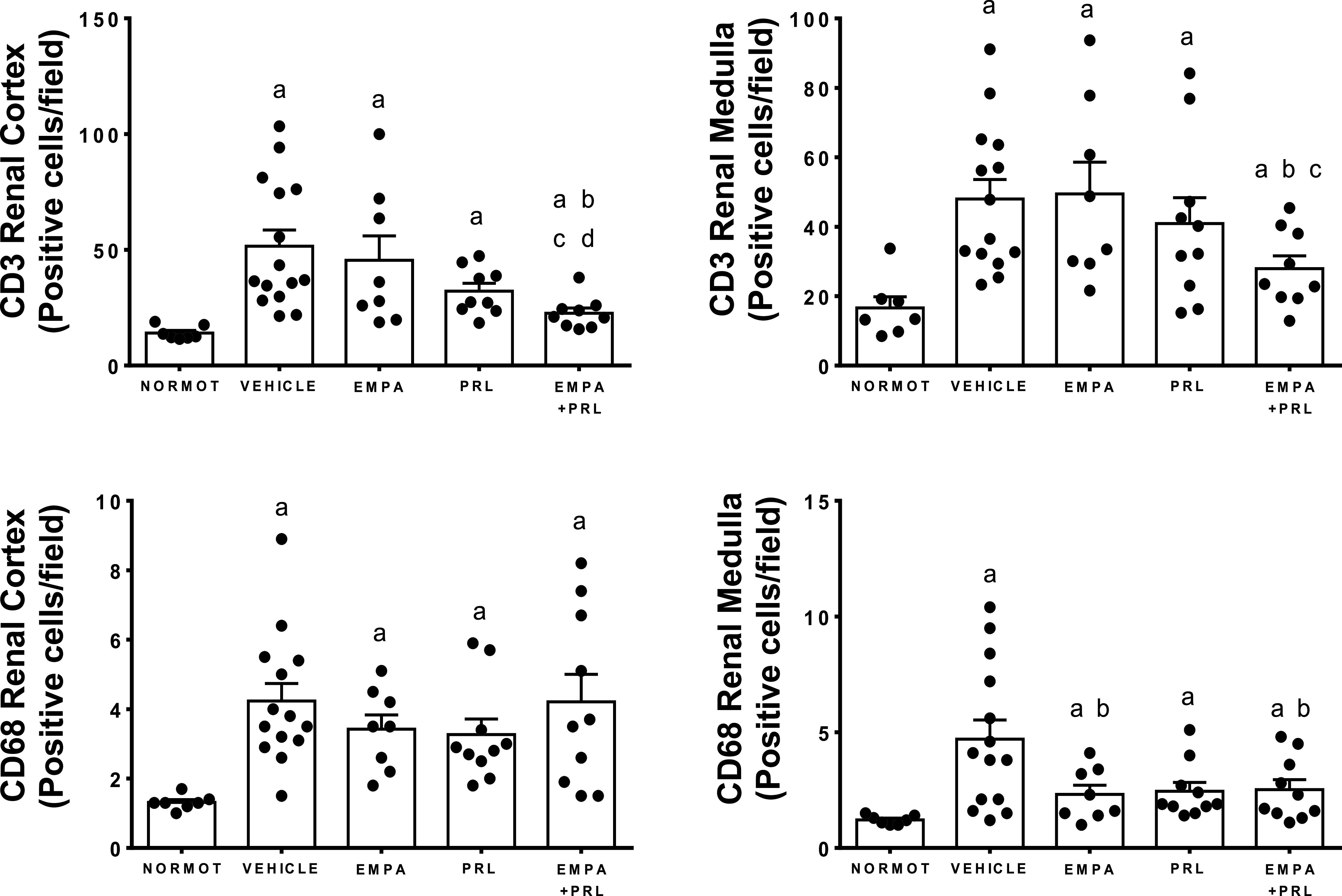
T-CD3 lymphocytes and CD68 macrophages count in the renal cortex and outer medulla in normotensive rats (NORMOT) (*n* = 7), and in HFD-fed hypertensive rats treated with vehicle (*n* = 14 to 15), empagliflozin (EMPA) (*n* = 8), praliciguat (PRL) (*n* = 9 or 10) or empagliflozin + praliciguat (EMPA+PRL) (*n* = 9 or 10). Values are shown as means ± SE. Differences between groups were assessed with the use of unpaired *t* test. ^a^*P* < 0.05 vs. normotensive; ^b^*P* < 0.05 vs. vehicle; ^c^*P* < 0.05 vs. empagliflozin; and ^d^*P* < 0.05 vs. praliciguat.

**Figure 11.**
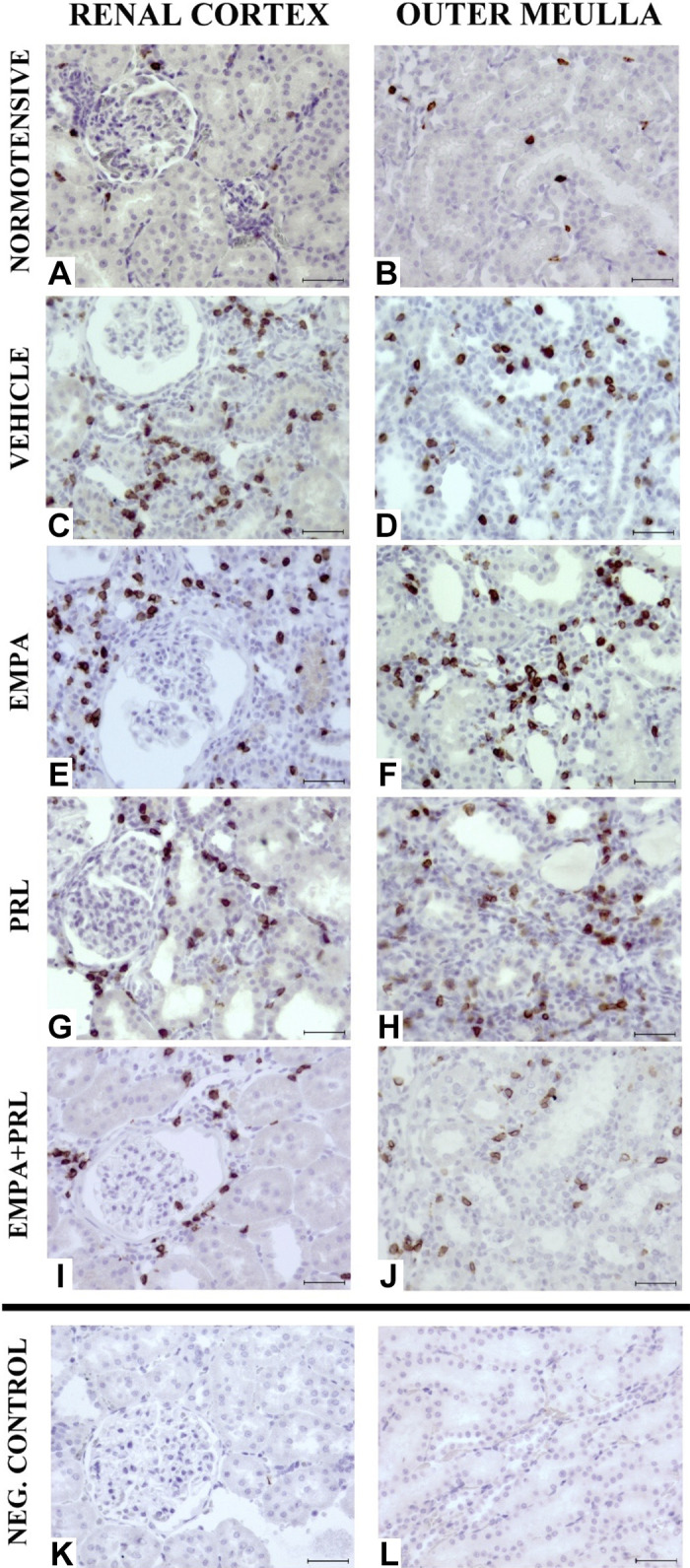
Representative images of T-CD3 lymphocyte infiltrate in the renal cortex and outer medulla in 19 wk old normotensive rats (*A* and *B*) and high-fat diet (HFD)-fed hypertensive rats treated from 12 wk of age with vehicle (*C* and *D*), empagliflozin (EMPA) (*E* and *F*), praliciguat (PRL) (*G* and *H*) or EMPA + PRL (*I* and *J*). Negative controls (*K* and *L*) were used to discard false positive immunolabelling. Indirect ABC anti-T-CD3 antigen. ×400. Scale bar: 50 μm.

The renal infiltration of immune cells was greater (*P* = 0.001) in the renal cortex of vehicle-treated hypertensive rats (T-CD3: 52 ± 7 positive cells/field; CD68: 4.2 ± 0.5 positive cells/field) than of normotensive rats (T-CD3: 14 ± 1 positive cells/field; CD68: 1.3 ± 0.1 positive cells/field). The infiltration was also more pronounced (*P* = 0.001) in the renal medulla of vehicle-treated hypertensive rats (T-CD3: 48 ± 6 positive cells/field; CD68: 4.7 ± 0.8 positive cells/field) than of normotensive rats (T-CD3: 17 ± 3 positive cells/field; CD68: 1.2 ± 0.1 positive cells/field) (Fig. 10). Representative images of T-CD3 lymphocytes infiltration in each group are shown in Fig. 11. EMPA or PRL administration did not induce changes of the T-CD3 cells infiltration in the renal cortex or renal medulla. However, fewer T-CD3 cells were detected in the renal cortex (23 ± 2 positive cells/field, *P* = 0.0007) and renal medulla (28 ± 4 positive cells/field, *P* = 0.016) of rats treated with EMPA+PRL (Fig. 10). The infiltration of CD68 macrophages in the renal cortex was similar in rats treated with vehicle, EMPA (3.4 ± 0.4 positive cells/field), PRL (3.3 ± 0.5 positive cells/field), or EMPA+PRL (4.2 ± 0.8 positive cells/field). However, the infiltration of CD68 macrophages was lower (*P* < 0.05) in the renal medulla of rat treated with EMPA (2.3 ± 0.4 positive cells/field, *P* = 0.049), or EMPA+ PRL treatment (2.5 ± 0.4 positive cells/field, *P* = 0.048) than in vehicle-treated rats (Fig. 10).

## DISCUSSION

This study provides the first exploration of whether the prolonged administration of a sGC stimulator or a SGLT2 inhibitor improves the cardiovascular, renal, and metabolic dysfunctions induced by an early and prolonged exposure to HFD when nephron endowment is reduced. It is also the first study evaluating whether the simultaneous stimulation of sGC and inhibition of SGLT2 have greater effects than the administration of either one alone. The results obtained show that SGLT2 inhibition potentiates the beneficial cardiovascular, renal, and metabolic effects elicited by sGC stimulation because the effects of the simultaneous administration of PRL and EMPA are greater than those elicited by either PRL or EMPA alone. The effects of the simultaneous treatment may be related to a greater reduction in the inflammatory status.

The increase in SAP elicited by the prolonged HFD in rats with ARDev had already been reported by our group (2) and is like that induced by HFD in rats with ARDev induced by prenatal dexamethasone (1). Prolonged exposure to HFD from an early age results in a further reduction of renal functional reserve in rats with ARDev and exposes the glomerulus to elevated renal perfusion pressures, with the consequent glomerular damage, the impairment of renal function and the exacerbation of hypertension (2). This study examines to what extent modulating NO/sGC pathway attenuates the cardiovascular, renal, and metabolic effects of a prolonged HFD in rats with ARDev whose hypertension and deterioration of renal function are associated with a decrease in eNOS bioavailability and increases in oxidative and inflammatory markers (2, 4). It is also known that impaired eNOS activity is associated with glucose intolerance and insulin resistance (6). The results obtained confirm the relevance of the NO/sGC pathway because SAP, TPR, body weight, FAV, and leptin levels were lower in hypertensive rats treated with PRL than in those treated with vehicle. Renal function was also improved in hypertensive rats treated with PRL. The decrease of AP in hypertensive rats with HFD treated with PRL is consistent with its lowering AP effect in SHR and Dahl salt-sensitive hypertensive rats (11) and may be related to a reduction of ANG II effects, partly mediated by a decrease in oxidative stress and inflammatory mediators. This possibility is supported by our results showing that PRL induced a decrease of AP that is like that observed after the administration of an AT_1_ receptor antagonist (2). A decrease of SAP after sGC stimulation or blocking ANG II effects has also been reported in hypertensive Dahl Salt-sensitive rats (12). It has also been shown that NO modulates the ANG II vascular effects (27) and that the AP changes induced by an AT_1_ receptor antagonist are mediated by a reduction in oxidative stress and inflammatory pathways (2).

Contrary to what was found in response to sGC stimulation, EMPA administration did not induce a significant change of SAP in rats with ARDev and prolonged HFD. This SAP response is in contrast with the decrease of AP elicited by EMPA in obese rats with high sodium intake (12) but is like that described in diabetic Dahl salt-sensitive rats (28) and in patients with type 2 diabetes (29). However, the simultaneous administration of EMPA amplified the effect of PRL on AP. Considering that sodium excretion only increased in hypertensive rats treated with EMPA and PRL, the earlier fall in AP with both treatments may reflect the combined effect of EMPA to reduce plasma volume acting together with the stimulation of sGC. Regarding cardiac function, our results show that PRL treatment led to a significant increase in SV and CO. These cardiac effects can likely be attributed to the observed AP reduction and may also be attributed to a direct cardiac effect of PRL since long-term sGC stimulation improves heart function, in part through an antifibrotic effect (10). The beneficial effects of PRL in improving cardiac function have also been shown in several models of heart disease (21).

The metabolic dysfunction in hypertensive rats with ARDev and prolonged HFD is evident because these rats have an increase in body weight and FAV, and elevated leptin and TG levels. Moreover, these rats were prediabetic since they have significantly elevated plasma insulin levels, HOMA-IR, and insulin resistance. The enhanced deleterious metabolic response to a HFD has also been reported in rats with intrauterine growth restriction (IUGR) and ARDev (3). Our results confirm previous findings illustrating that pharmacological stimulation of sGC can have favorable effects on metabolism (9, 30). PRL administration led to a reduction of body weight gain and plasma levels of leptin and TG, possibly attributable to the decrease in FAV, due to an increase in whole body energy expenditure (9). The decrease in leptin may contribute to the beneficial cardiovascular effects found during PRL treatment. However, the precise mechanisms driving a decrease in leptin in the absence of changes in food intake need to be further examined.

Evaluating the metabolic effects of a SGLT2 inhibitor in rats with a reduced nephron endowment is relevant because the effects induced by SGLT2 inhibition has been proposed to be dependent of the number of intact nephrons (16). In contrast to the metabolic effects elicited PRL, EMPA only led to a decrease in the plasma glucose and TG levels that could contribute to the lower body weight gain. A metabolic effect of SGLT2 inhibition has only been reported in diabetic rats (29). An important new finding of this study is that the metabolic effects of sGC stimulation are potentiated by SGLT2 inhibition since the simultaneous PRL and EMPA treatment, but not the administration of either one alone, improved glucose tolerance and reduced plasma insulin and HOMA-IR to the levels detected in normotensive rats. In addition, the body weight gain was lower in rats treated with PRL and EMPA and the effects of their combined administration on FAV and plasma leptin concentration tend to be consistently greater, albeit not significantly so, than those elicited by PRL alone. The absence of changes in plasma adiponectin concentration in rats treated with EMPA and/or PRL was unexpected since a reduction in body weight is typically associated with an increase of this adipokine (31).

The renal changes following prolonged exposure to HFD and a reduced nephron endowment have been reported by our group (2) and are similar to those elicited by a HFD in IUGR elicited by a maternal placental insufficiency (32). However, little is known regarding the effects of sGC stimulation on renal function in hypertension secondary to an ARDev and a prolonged HFD. The hypothesis was that sGC stimulation would improve renal function because hypertensive rats with ARDev have a decrease in the eNOS expression (4) and an increment in oxidative stress (5). The increase in creatinine clearance and decrease in plasma urea concentration without changes in RBF associated with PRL treatment, despite a significant reduction in AP, suggest the involvement of an altered NO-cGMP pathway in the deterioration of renal function in rats with ARDev and HFD. These results are consistent with those showing that PRL administration leads to reductions in inflammatory cytokine levels, proteinuria, and renal fibrotic gene expression in Dahl salt-sensitive hypertensive rats (11, 22), and that the improvement of renal damage occurs at doses of PRL having minimal effects on AP pressure (22). It has also been reported that sGC activation induces an AP reduction and an increase of GFR in Dahl salt-sensitive rats (33).

The reno-protective effect of SGLT2 inhibitors has been reported in models of diabetes-related hyperfiltration (28) and in mice with Western diet-induced obesity (11), but it was unknown whether they influence renal hemodynamic in hypertensive rats with reduction in renal function reserve and HFD. The results obtained illustrate that treatment with EMPA alone induces an increase in glucosuria and improves GFR, estimated by creatinine clearance, without lowering AP. The mechanisms involved in this renal response are unknown, but it is conceivable that prolonged administration of SGLT2 inhibitors may induce a deterioration of renal function in rats with ARDev and HFD because AP remained elevated. In the current study, the combined treatment with PRL and EMPA leads to an improvement of GFR and a decrease in AP and RVR, without changes in RBF. As already mentioned, the higher UNaV observed in rats treated with both PRL and EMPA may have contributed to the earlier decrease of AP found in rats treated with PRL and EMPA than in rats only treated with PRL. A differential effect on Na^+^ versus glucose excretion was found in rats treated with EMPA alone or EMPA+PRL. The effect of EMPA alone on glucose but not on Na^+^ excretion could be explained by an increase of Na^+^ reabsorption in distal tubular segments that could compensate the proximal tubular effect of EMPA. However, this possible compensation in distal tubular segments probably does not occur in rats treated with EMPA+PRL because it has been shown that an increase in NO, with the consequent activation of sGC, reduce Na^+^ reabsorption in proximal and distal tubular segments (19).

An important reno-protective effect of this simultaneous treatment is the significant decrease in the infiltration of T-CD3 lymphocytes in the renal cortex and renal medulla that were not found when PRL or EMPA were administered alone. Fewer infiltrating T-CD3 lymphocytes and resulting lower levels of inflammatory cytokines and oxidative stress, would be expected to contribute to a further improvement of renal function (33). However, treatment with PRL and EMPA did not attenuate the enhanced macrophages infiltration observed in hypertensive rats with ARDev and HFD. These results are in contrast with those showing that the administration of a sGC stimulator reduces macrophages infiltration in the liver of HFD-induced obese mice (34) and in kidneys of rats with an acute form of glomerulonephritis (35). The mechanisms implicated in the greater renal beneficial effects induced by the simultaneous administration of PRL and EMPA remain unknown but an improvement in insulin resistance and a reduction in leptin and renal inflammatory cytokine levels could be involved.

One limitation of this study is that the effects of EMPA, PRL, or EMPA+PRL were not examined in normotensive rats. However, in studies in human healthy subjects, the sGC stimulator PRL decreased BP (36) and the SGLT2 EMPA induced a dose-dependent glucosuria without other clinically safety concerns (37). In addition, future studies need to examine whether the cardiovascular and renal effects of the simultaneous administration of EMPA and PRL are accompanied by a reduction in glomerular injury, as well as by changes in the molecular levels on genes and proteins of fibrosis and inflammation.

Together, the results of this study may have pathophysiological implications because it is illustrated that the combined administration of a sGC stimulator and a SGLT2 inhibitor leads to a greater improvement of the cardiovascular, renal, and metabolic dysfunctions secondary to an ARDev and prolonged exposure to HFD than the administration of only a sGC stimulator or a SGLT2 inhibitor. However, it remains to be examined whether there are sex-dependent differences in the cardiovascular, renal, and metabolic effects of the combined administration of EMPA and PRL. We studied male rats because the cardiovascular and renal changes and the increments in oxidative stress, glomerulosclerosis and renal infiltration of CD3 lymphocytes associated to a prolonged HFD in hypertensive rats are greater in males than in females (2). The metabolic response in females may be different than that found in males since the cardiovascular risk conferred by metabolic syndrome is highly dependent on sex and sex hormone status (38). Future studies examining whether there are sex-dependent differences are relevant because “understanding individualized metabolic syndrome based on sex is of immense clinical importance to population-wide cardiovascular risk” (38).

## GRANTS

This work was partly supported by the Instituto de Salud Carlos III, Ministerio de Ciencia e Innovación Grant PI16/01556 (cofunded by European Regional Development Fund/European Social Fund “A way to make Europe/Investing in your future”) and Fondos FEDER.

## DISCLOSURES

C. Shea, C. Schwartzkopf, E.S. Buys, and J.L. Masferrer were full-time employees of Cyclerion when the experiments of this study were performed and may own stock options in Cyclerion Therapeutics. V. Reverte, F. Rodriguez, L. Oltra, J.M. Moreno, M.T. Llinás, and F.J. Salazar do not have any financial or personal association with Cyclerion Therapeutics other than the funding and collaboration for this project and had full control over the design of the study, the decision to publish, and the content of the manuscript. None of the other authors has any conflicts of interest, financial or otherwise, to disclose.

## AUTHOR CONTRIBUTIONS

C.M.S., J.L.M., and F.J.S. conceived and designed research; V.R., F.R., L.O., J.M.M., M.T.L., C.M.S., C.D.S., and F.J.S. performed experiments; V.R., F.R., J.M.M., M.T.L., C.D.S., E.S.B., J.L.M., and F.J.S. analyzed data; V.R., F.R., J.M.M., M.T.L., C.M.S., C.D.S., E.S.B., J.L.M., and F.J.S. interpreted results of experiments; V.R. and F.R. prepared figures; V.R. and F.J.S. drafted manuscript; V.R., F.R., L.O., J.M.M., M.T.L., C.M.S., C.D.S., E.S.B., J.L.M., and F.J.S. edited and revised manuscript; V.R., F.R., L.O., J.M.M., M.T.L., C.M.S., C.D.S., E.S.B., J.L.M., and F.J.S. approved final version of manuscript.
